# Optimization of leaf morphology in relation to leaf water status: A theory

**DOI:** 10.1002/ece3.6004

**Published:** 2020-01-22

**Authors:** Junyan Ding, Edward A. Johnson, Yvonne E. Martin

**Affiliations:** ^1^ Biogeoscience Institute University of Calgary Calgary Alberta Canada; ^2^ Department of Biological Sciences University of Calgary Calgary Alberta Canada; ^3^ Lawrence Berkeley National Laboratory Berkeley CA USA; ^4^ Department of Geography University of Calgary Calgary Alberta Canada

**Keywords:** hydraulic traits, leaf carbon budget, leaf shape, safety–efficiency trade‐off, stomatal optimization, vascular system, vein structure, xylem resistance

## Abstract

The leaf economic traits such as leaf area, maximum carbon assimilation rate, and venation are all correlated and related to water availability. Furthermore, leaves are often broad and large in humid areas and narrower in arid/semiarid and hot and cold areas. We use optimization theory to explain these patterns. We have created a constrained optimization leaf model linking leaf shape to vein structure that is integrated into coupled transpiration and carbon assimilation processes. The model maximizes net leaf carbon gain (NPP_leaf_) over the loss of xylem water potential. Modeled relations between leaf traits are consistent with empirically observed patterns. As the results of the leaf shape–venation relation, our model further predicts that a broadleaf has overall higher NPP_leaf_ compared to a narrowleaf. In addition, a broadleaf has a lower stomatal resistance compared to a narrowleaf under the same level of constraint. With the same leaf area, a broadleaf will have, on average, larger conduits and lower total leaf xylem resistance and thus be more efficient in water transportation but less resistant to cavitation. By linking venation structure to leaf shape and using water potential as the constraint, our model provides a physical explanation for the general pattern of the covariance of leaf traits through the safety–efficiency trade‐off of leaf hydraulic design.

## Graphical abstract

A leaf model integrates venation and leaf shape in coupled photosynthesis and transpiration processes to maximize carbon assimilation rate. Model predicts a general increase in trend‐leaf size and net leaf carbon gain with water status by coadjustment of stomatal and xylem resistance. With same water status, narrower leaf has lower net carbon gain but larger number of smaller conduits indicating safety‐efficiency trade‐off.

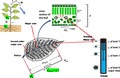

## 1 INTRODUCTION

Understanding how plants adapt to different physical environments is one of the central themes of plant ecology. As the fundamental production units of plants, leaves have presumably evolved to maximize net carbon gain (Blonder, Violle, Bentley, & Enquist, 2011; Chabot and Hicks, 1982; Westoby, Falster, Moles, Vesk, & Wright, 2002; Williams, Field, & Mooney, 1989), which ultimately affects plant fitness (Shipley, Lechowicz, Wright, & Reich, 2006; Kikuzawa, 1995; Wright et al., 2004). Empirical studies have found a correlation between several leaf traits that are believed to comprise the adaptive strategy that affects leaf economy, known as the leaf economic spectrum (Wright et al., 2004). Furthermore, leaf surface area has been found to be positively associated with soil moisture levels (Wright et al., 2017; Xu, Guo, Xu, Wei, & Wang, 2009), and net carbon assimilation rate and leaf area are found to decrease toward the top of a tree, with decreasing petiole xylem water potential (Ambrose, Sillett, & Dawson, 2009; Ishii, Jennings, Sillett, & Koch, 2008; Kenzo et al., 2015; Koch, Stillet, Jennings, & Davis, 2004; Ryan, Phillips, & Bond, 2006). This phenomenon suggests that water potential can also affect leaf shape. Most previous studies on leaf traits have focused on thermal regulation; less attention has been paid to the leaf water status. The purpose of this paper is to propose a mechanism for the leaf economic spectrum in relation to water status.

The leaf water status directly affects the leaf carbon budget through a series of biochemical processes. When the water potential of the leaf vein conduit reaches a critical low value, cavitation occurs. Cavitation in minor vein conduits blocks their ability to fill with water and causes the loss of cell turgor pressure, which in turn negatively affects the biochemical processes of photosynthesis by reducing the maximum carboxylation rate, damaging ATP, and increasing mesophyll resistance for CO_2_ diffusion (Grassi & Magnani, 2005; Lawlor & Tezara, 2009; Niinemets, Cescatti, Rodeghiero, & Tosens, 2005). Leaf traits considered to control the leaf carbon budget related to water status include stomatal conductance, leaf area, leaf shape, and venation structure (Ball, Woodrow, & Berry, 1987; Berninger, Mäkelä, & Hari, 1996; Blonder et al., 2011; Cowan, 1982, 1986; Jarvis, 1976; Noblin et al., 2008; Wright et al., 2017; Xu et al., 2009).

Stomatal conductance and leaf area are the two fundamental leaf traits that are tightly associated with the leaf carbon budget, the leaf water status, and water loss. It has been shown that to avoid the negative impacts of low water potential, the leaf will increase its stomatal resistance to reduce the transpiration rate (Ball et al., 1987; Berninger et al., 1996; Cowan, 1982, 1986; Jarvis, 1976). Stomatal resistance can be regulated by instantaneously changing stomata size (opening and closure) and varying the stoma density of the leaf over a longer time scale (Sack & Buckley, 2016; Woodward & Kelly, 1995; Xu & Zhou, 2008). Carbon and water fluxes are coupled through stoma. This adjustment comes at the cost of a reduced carbon fixing rate, since increasing stomatal resistance slows the carbon intake rate. Leaf surface area has been found to be positively associated with soil moisture levels (Wright et al., 2017; Xu et al., 2009). Furthermore, the net carbon assimilation rate and leaf area are found to decrease with tree height, because of the decreasing xylem water potential associated with tree height (Ambrose et al., 2009; Ishii et al., 2008; Kenzo et al., 2015; Koch et al., 2004; Ryan et al., 2006). These studies suggest that the adjustment of leaf area to water availability over time can influence the optimization of stomatal resistance in response to fluctuations in temperature and vapor pressure (Maseda and Fernández, 2006).

Leaf shape and leaf venation are the two other important traits relating to the leaf carbon budget and water use strategy. The former determines external geometry, and the latter determines the structure of the internal transportation system. Both can affect the rate at which net leaf carbon gain (NPP_leaf_) scales with leaf size. Leaf shape, a genetically determined trait, is found to be associated with habitat and is a trait that defines plant water use strategies. Leaf width is found to increase with increasing precipitation and/or soil moisture (Givnish, 1987; McDonald, Fonseca, Overton, & Westoby, 2003). Plants growing in arid or semiarid environments have smaller and narrower leaves, while plants growing in humid or semihumid environments have broader and larger leaves (Wright et al., 2017). The leaf venation network has been connected to net carbon assimilation rate, life span, leaf mass per area, and nitrogen content, the traits that affect the leaf carbon budget (Blonder et al., 2011).

Evidence suggests that leaf shape and venation structure are related. To provide structural support for a leaf, the major vein must extend from the petiole to the end of the leaf, and second‐order major veins must extend from the major vein to the boundary of the leaf (Cochard, Nardini, & Coll, 2004; Roth‐Nebelsick, Uhl, Mosbrugger, & Kerp, 2001). Therefore, assuming the venation has a dendritic structure following the metabolic scaling theory (Price & Enquist, 2007; West, Brown, & Enquist, 1997), the width‐to‐length ratio of a leaf regulates the vein network parameter γ (the ratio of the length of *n* + 1th order vein to nth order vein). Thus, a narrowleaf will have a smaller width‐to‐length ratio, and the consecutive veins will consequently shorten more quickly than those of a broadleaf (e.g., with the same leaf length, the second‐order major vein of a narrowleaf will be shorter than that of a broad leaf). In addition, the optimal average distance between the two adjacent smallest minor veins approximates the distance from the minor vein to the leaf surface, which is approximately half of the leaf thickness (Noblin et al., 2008). These venation properties control the rate of total leaf xylem resistance scaling with leaf area (West, Brown, & Enquist, 1999).

The studies reviewed above suggest that venation structure, leaf shape, and stomatal resistance are all related and affect the trade‐offs between leaf size and the characteristics of the conductive system in leaves and transpiration. The total carbon uptake and the construction and maintenance costs of a leaf are proportional to leaf size. Therefore, these leaf traits affect leaf carbon budget and leaf water status through transpiration. The objective of this study is to develop a leaf physiology model to explore how the leaf deals with this trade‐off to maximize its net carbon gain.

Optimality is suggested as an appropriate method to model the adaptation strategy of plants to their environments (Schymanski, Sivapalan, Roderick, Beringer, & Hutley, 2008). Recently, optimality has been used to predict the instantaneous response of stomatal conductance to environmental conditions such as variation of soil moisture and elevated atmospheric CO_2_ concentration (Ball, Cowan, and Farquhar, 1988; Cowan, 1982; Franks and Casson, 2014; Manzoni et al., 2013; Medrano et al., 2015; Sperry et al., 2016, 2017). These models optimize instantaneous stomatal conductance either by water loss or the loss of plant hydraulic conductance (Sperry et al., 2016, 2017) without explicitly considering the effect of other leaf properties, such as size, shape, and venation structure.

Here, we use an optimality approach to explain the coadjustment of leaf traits as an adaptive strategy in a given environment. We present a constrained optimization leaf model that incorporates leaf venation and shape with coupled transpiration and carbon budget processes to address the coadjustment of these two leaf traits as an adaptive strategy. As mentioned above, stomatal conductance can be regulated through two means: the instantaneous opening and closing of stoma and the long‐term adjustment of stoma density and aperture size. Our model focuses on the latter. Here, our model is not an optimization model of instantaneous stomatal conductance, in the sense that it does not accurately resolve the transpiration rate under temporally detailed realistic conditions; instead, the model provides an explanation of the adaptation strategy in an environment over the life span of the leaf rather than predicting/explaining the instantaneous response of stoma. The model also provides a physical link between stoma behavior and other leaf traits. Furthermore, our model uses water potential as the limiting factor, instead of water, as used in previous models (see Section 2.1 for justification).

## 2 CONSTRAINED OPTIMIZATION LEAF MODEL

In general, a constrained optimization model has two components (functions): an objective function, describing the profit (net carbon gain) of a system (leaf) to be maximized, and a constraint function, defining the total cost of a factor allowed to make profit. Here, the constraint is the maintenance of the loss of water potential from the petiole to the terminal minor vein above a critical value, which depends on the petiole water potential. This is somewhat similar to imposing a hydraulic risk avoidance that works at the leaf level (i.e., without a description of the decrease in water potential from soil to petiole).

Below, we first justify the appropriateness of using water potential as the constraint factor and then describe the two components of the model: the objective function and the constraint function.

### 2.1 Constraint: water or water potential

A critical issue with the optimality approach is the appropriate structuring of the model (Sperry et al., 2017; Wolf, Anderegg, & Pacala, 2016). Available water in soil has long been considered the limiting factor of plant growth. For example, the stomatal optimization theory assumes that the adjustment of stomatal resistance leads to the maximization of the cumulative carbon assimilation over a fixed loss of water through transpiration (Cowan & Farquhar, 1977, Katul, Oren, Manzoni, Higgins, & Parlange, 2012, Sperry et al., 2017).

However, in some regions (e.g., humid areas and topographic convergent areas), water is not limited. Furthermore, in places where plants can access groundwater, water itself is not limited, because no matter how much water the plant uptakes from the capillary fringe, it can be recharged from groundwater in the saturated zone. As such, water cannot be used as limiting factor in the optimization model for these regions. However, a cap of root zone water potential exists, because roots do not grow in the saturated zone, whether because it is biologically imperative to avoid anoxic conditions or because roots will physically rot if they are in water too long. Also, it is the lowering of water potential of the xylem that causes the cavitation and negative impact on plants. Therefore, the amount of water potential a plant can lose through transpiration is limited, and this limitation is valid not only in arid regions but also in humid ones. By using water potential as the limiting factor, optimization can be applied in these regions.

In addition, average root zone water potential is not solely controlled by precipitation—the input of water, but also the soil depth and the depth of groundwater. Thus, using water as the limiting factor cannot consider all of these factors, but using water potential does allow us to further integrate these aspects in the future. Furthermore, several leaf economic traits that vary with soil moisture are found globally. This indicates that water can regulate plant behavior not only in arid regions but also in humid areas. As such, using water potential, instead of water, as the limiting factor in the optimization model not only allows the optimization model to be valid for all regions but also holds the potential to integrate the processes of hillslope hydrology in the future. So, for all of these reasons, we use water potential as an alternative constraint.

### 2.2 Constrained optimization leaf model

The constrained optimization leaf model has two components: an objective function, as the net leaf carbon gain (NPP_leaf_ *)*, and a constraint function, the total loss of xylem water potential of a vein, starting at the petiole and terminating at the minor vein. The model maximizes the objective function, the NPP_leaf_, subject to the constraint function, the total loss of xylem water potential of a vein (petiole xylem water potential) over a leaf's life span.

#### 2.2.1 The objective function—net leaf carbon gain

NPP_leaf_ is described here as the balance between the leaf carbon assimilation rate (the first term on the right side of Equation 1) and the rate of total carbon loss from respiration and construction over its life span (the second term on the right side of Equation 1). The leaf carbon assimilation rate is the gross primary productivity rate, or the net carbon fixation rate per unit of leaf area, GPP (μmol m^−2^ s^−1^), multiplied by the leaf area of a single leaf, *LA* (m^2^):

(1) $$\begin{array}{cc} {\text{NPP}}_{\text{leaf}} & =\text{GPP}\cdot \text{LA}-R\cdot {\text{LA}}^{3/2}\\ \text{with GPP} & =\frac{C_{a}\cdot a_{1}}{1.6\cdot \text{cons}3\cdot a_{1}\cdot r_{\mathrm{es}}+\left(a_{2}+S\cdot C_{a}\right)} \end{array}$$

where NPP_leaf_ (mol/s) is the net carbon gain of a mature leaf. *R* (mol m^−1^ s^−1^) is the coefficient of the total carbon cost of the leaf (the second term on the right side of Equation 1), the sum of the life span standardized construction cost (e.g., the total mass of the leaf divided by its life span) and the respiration loss, both are proportional to total mass of a leaf. The total mass is the product of the average leaf mass density and the volume of the leaf. Assuming the average density of the leaf does not change with leaf size, for a given leaf geometry, the total carbon cost of the leaf scales with leaf area, by an exponent of 3/2. LA (m^2^) is the leaf area; *C_a_* (0.0004 mol/mol) is the CO_2_ concentration of air; *a*_1_ (mol m^−2^ s^−1^) is the maximum carboxylation capacity; and *a*_2_ (510 mol/mol) is a given as *K_c_* (1 + Co*_a_*/*K_o_*), where *K_c_* and *K_o_* are the Michaelis constants for CO_2_ fixation and oxygen inhibition, and Co*_a_* is the oxygen concentration in the air. *r*_es_ (s/m) is the mass stomatal resistance of water vapor; cons3 (0.025 m^3^/mol) is a constant for stomatal resistance conversion from mass to mol; *S* is the ratio of leaf CO_2_ concentration to the CO_2_ concentration of the air, which is considered a constant with a value around 0.6 (Katul, Manzoni, Palmroth, & Oren, 2010).

The formula of GPP used here is adapted from the linear model of Katul et al. (2010) at equilibrium. The linear model assumes that the ratio of inner leaf CO_2_ concentration, *C_i_*, to the CO_2_ concentration of the air in the denominator of the biochemical model is a constant, while allowing *C_i_* to vary in the numerator (Katul et al., 2010). Although this linear model will slightly overestimate GPP, it provides a reasonable approximation of the more realistic nonlinear model (Katul et al., 2010; Vico, Manzoni, Palmroth, Weih, & Katul, 2013). We use the linear model so that we can obtain a relatively simple and understandable expression.

#### 2.2.2 The constraint function—the total loss of xylem water potential (Ψ_loss_)

The constraint function describes the total loss of xylem water potential from the petiole to the terminal minor vein under the limitation that the total loss of xylem water potential is the product of the average mass flow rate per unit area of cross section of xylem, *Jx (*m^3^ s^−1^ m^−2^ *)*, and the total xylem resistance per unit area of the cross section of a single flow path, *r*_Xtotal_ (s/m) (see Appendix S1 for derivation):

(2a) $$\begin{array}{cc} \Psi_{\text{loss}} & =J_{x}\cdot r_{X\text{total}}\\ \;J_{x} & =\frac{e_{\text{sat}}-e_{a}}{\frac{\frac{k_{z}\cdot Z}{2}\cdot e_{\text{sat}}}{\text{cons}1\cdot D_{m}\cdot w_{0}}+\frac{r_{\mathrm{es}}}{\text{cons}2}}\cdot \frac{k_{z}\cdot Z\cdot l_{T}}{2\pi \cdot r_{T}^{2}}\\ \;r_{X\text{total}} & =\frac{8\cdot \mu }{\rho_{w}}\cdot \frac{l_{T}}{r_{T}^{2}}\cdot \frac{1-\frac{\beta^{2}}{\gamma }\cdot {\left(\frac{l_{T}}{{\left(\text{LA}/k_{\text{SP}}\right)}^{0.5}}\right)}^{\frac{\ln(\beta^{2}/\gamma )}{\ln\gamma }}}{1-(\beta^{2}/\gamma )} \end{array}$$

(2b) $$\Psi_{\text{loss}}\leq \Delta \Psi_{L\max},\;\Delta \Psi_{L\max}=\Psi_{P}-\Psi_{C}$$

where Ψ_loss_ (Pa) is the total loss of xylem water potential from the petiole to terminal minor vein; *e*_sat_ is the saturated vapor pressure; *e_a_* is the atmospheric vapor pressure; cons1 (kg/mol) is the molar mass of H_2_O; cons2 (s^2^/m^2^) is the inverse of the specific gas constant of water vapor; *k_Z_* is the empirical scaling coefficient providing the ratio of minor vein distance to leaf thickness; *Z* (m) is the thickness of the leaf; *r_X_*_total_ is the total xylem resistance of a single flow path from petiole to terminal minor vein; *D_m_* is the diffusivity of water in mesophyll; *w*_0_ (mol/m^3^) is the saturated water concentration of mesophyll; *l_T_* (m) is the length of the terminal minor vein; *r_T_* (m) is the radius of the terminal minor vein xylem conduit; µ (kg m^−1^ s^−1^) is the viscosity of water; *ρ_w_* (km/m^3^) is the density of water; *k*_SP_ is the shape parameter of the leaf (proportional to *W/L* ratio); *γ* and *β* are the parameters of the venation network (explained in the next paragraph); and ΔΨ*_L_*_max_ (Pa) is the maximum total xylem water potential that is allowed to be lost along a single flow path from petiole to terminal minor vein without causing any negative impact on leaf health. It is the difference between the petiole xylem water potential Ψ*_P_* (Pa) and the turgor loss xylem water potential Ψ*_C_* (Pa) at which the percentage of the vein that forms cavitation will cause detrimental effects on biochemical processes. Ψ*_C_* is the hydraulic trait of plants; thus, the value depends on the plant species. Ψ_50_ of the vulnerability curve of the sigmoid model (Markesteijn, Poorter, Paz, Sack, & Bongers, 2011; Pammenter & Willigen, 1998) will be a good estimation of Ψ*_C_*, because this is where the sharpest drop of conductance occurs (Venturas, Sperry, & Hacke, 2017).

**Figure 1:**
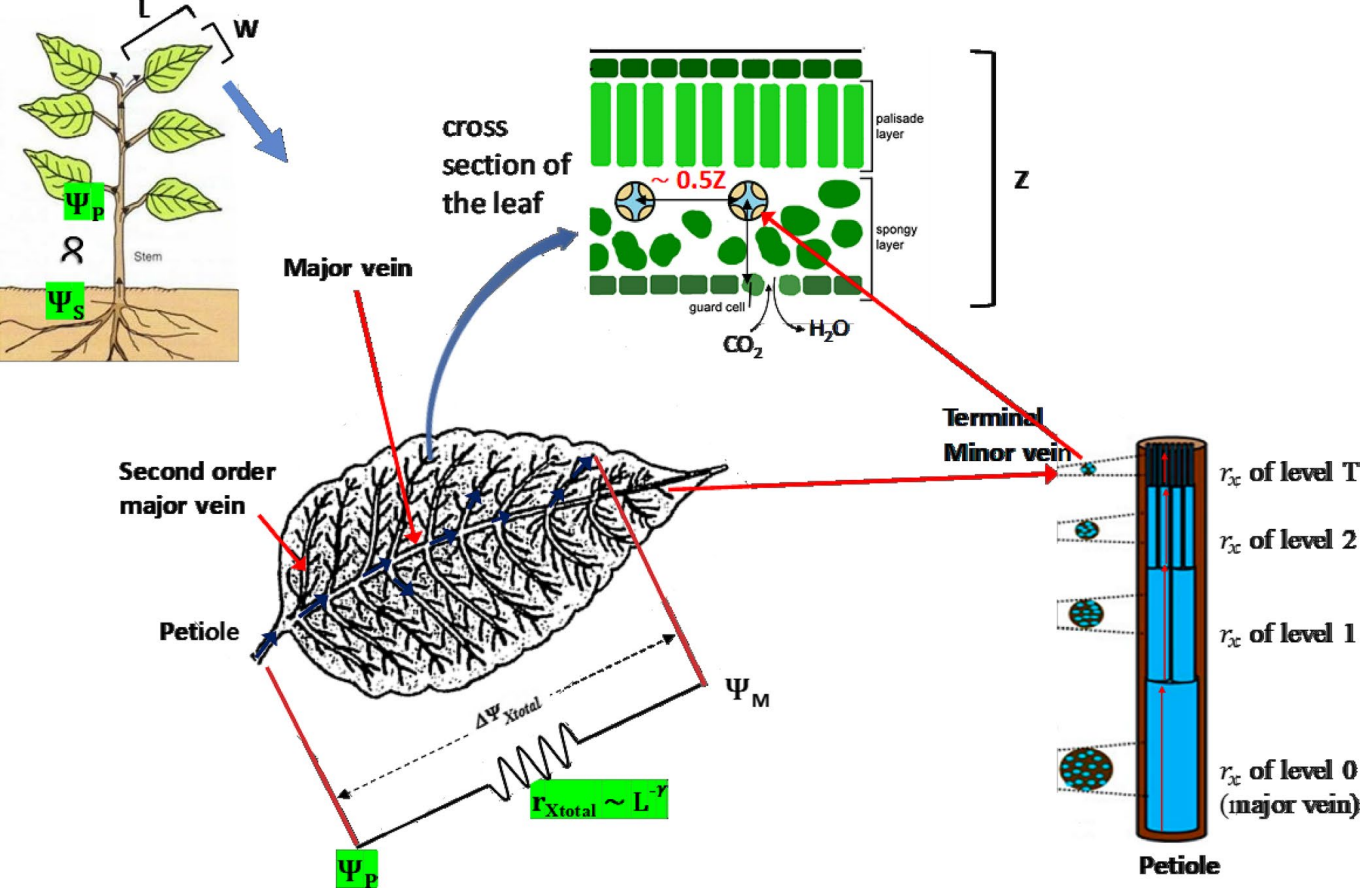
Schematic leaf model

The total xylem resistance is derived from a simplified hydraulic network structure following the metabolic scaling theory (Enquist et al., 2007). The vein network of our leaf model has a dendritic structure, as described by Price and Enquist (2007). The veins contain xylem conduits, and water flows through the xylem conduits contained by veins (Figure 1). Note that the vein network and hydraulic network composed by xylem conduits are not the same, but they do share some characteristics, described as follows. The veins are numbered in increasing order from major vein to terminal minor vein (e.g., the major vein is numbered as Order 0, the second major vein is numbered as Order 1, and so on). The xylem conduits branch where veins branch; therefore, the length ratio of two consecutive orders of veins and the length ratio of the two consecutive orders of xylem conduits are the same, which we denote as γ. However, the branching ratio of the vein and conduit differ. In our model, the branching ratio (the ratio of the number of conduits of two consecutive orders veins) of xylem conduit is denoted by *m*, and the vein branching ratio is denoted by *n.* The ratio of the radius of the two consecutive xylem conduits is denoted as *β*, assuming the conservation of total xylem conduit cross‐sectional area (Enquist et al., 2007; West et al., 1997), xylem conduit branching ratio, *m*, equals 1/*β* ^2^.

Based on the previous studies mentioned above, we make two connections between vein structure and leaf shape. First, the length ratio of two consecutive veins, γ, is proportional to half of the width‐to‐length ratio of the leaf. Second, the distance between two adjacent terminal minor veins is half of the leaf thickness (Figure 1).

If the water potential of the xylem conduits—which we call xylem water potential in the rest of the paper—of the terminal minor veins drops below a threshold (Ψ*_C_*), cavitation will occur, and thus leaf cell turgor pressure will no longer be maintained. The threshold xylem water potential (Ψ*_C_*) is a functional hydraulic trait determined by the vulnerability curve (Markesteijn et al., 2011; Pammenter & Willigen, 1998) and cell physiology. The difference between the actual petiole xylem water potential and the critical xylem water potential determines how much total water potential can be lost through the flow pathway.

From Equation 2, we can see that both increasing leaf area and decreasing stomatal resistance can increase NPP_leaf_, but they also increase the total loss of xylem water potential. From an evolutionary perspective, plants evolved in a given environment will tend to maximize NPP_leaf_ in that type of environment. Therefore, the model maximizes NPP_leaf_, the objective function (Equation 1), over the loss of total xylem water potential (Equation 2a), under the condition that the total loss of xylem water potential (Ψ_loss_) must be less than the difference between the petiole xylem water potential and the critical xylem water potential (Equation 2b).

Below, we explain in detail how leaf shape (*W/L* ratio) affects the network structure of the leaf hydraulic system and hence the difference in the relation between total xylem resistance and leaf area. The total number of xylem conduits of the major vein (0 Order vein) is the following:

(3) $$M_{0}=\frac{M_{T}}{m^{T}}$$

where *M_T_* is the number of xylem conduits of a single terminal minor vein, and m is the xylem conduits branching ratio (e.g., *M_k_*_+1_/*M_k_*). Following the metabolic scaling theory, the vein network is assumed to have a space‐filling, fractal structure (West et al., 1997). Thus, the total number of xylem conduits of the major vein, *M*_0_, scales to the power of the number of the total vein order, *T*, given by the following (see Appendix S1 for derivation):

(4) $$\frac{\ln\left(\frac{l_{T}}{{\left(\frac{\mathrm{LA}}{k_{\text{SP}}}\right)}^{0.5}}\right)}{\ln\left(\frac{1}{2}k_{\text{SP}}\right)}$$

Expanding Equation 4, *T* can be expressed as the following:

(5) $$T=\frac{-2\ln\left(l_{T}\right)-\ln\left(k_{\mathrm{SP}}\right)+\ln\left(\mathrm{LA}\right)}{2\ln\left(2\right)-2\ln\left(k_{\mathrm{SP}}\right)}$$

where *k*_SP_ = *W/L*, and *LA* is the total leaf area (m^2^). Both the numerator and denominator of Equation (5) decrease with increasing *W/L*, but the numerator decreases at a faster rate than the denominator. Therefore, *T* increases with increasing *W/L* ratio. *M_T_* is determined by leaf size, minor vein length, distance between minor veins, and number of xylem conduits contained by a single minor vein, assuming that the minor vein is invariant (e.g., similar in length and number of xylem conduits contained) and the minor vein distance is determined by leaf thickness. The number of minor veins is the total length of minor veins divided by the average length of a single minor vein, *l_T_*. Following the same logic of West et al. (1999) (see Appendix S1 for detail), the total length of minor veins is given as the leaf area divided by the average distance between two adjacent minor veins. Thus, with the same LA, *M_T_* is the same for leaves differing in *W/L* ratios if they have the same thickness. With a larger *T*, the number of xylem conduits of the major vein of a broadleaf is less than that of a narrowleaf (Equation 3). With the conservation of the total area of the cross section of xylem conduit, the radius of xylem conduit of the major vein, *r*_0_, of a leaf having larger *W/L* is larger than of a leaf with a smaller *W/L*. Therefore, if the leaf size is the same, and assuming the conduit size of terminal minor vein is the same, leaves with different shapes (*W/L* ratio) will have the same total cross section area of xylem conduits at petiole, but leaves with small *W/L* ratios will have fewer orders of veins and a larger number of smaller size xylem conduits of the major vein, compared to a leaf with a large *W/L* (Table 1).

**Table 1:** List of symbol

Symbol	Description	Units
*a*_1_	Plant biochemical parameter, in case of Rubisco limited photosynthesis, it is the maximum carboxylation capacity (*V_c_*_,max_ or *V_m_*)	μmol m^−2^ s^−1^
*a*_2_	Biochemical constant, for Rubisco limited case, *a*_2_ = *K_c_*(1 + Co*_a_*/*K_o_*), where *K_c_* and *K_o_* are the Michaelis constants for CO_2_ fixation and oxygen inhibition and Co*_a_* is the oxygen concentration in air	μmol/mol
*Ca*	CO_2_ concentration of air (ambient CO_2_ concentration)	mol/mol
*C_l_*	CO_2_ concentration within space of leaf	mol/mol
cons1	The molar mass of H_2_O (M(H_2_O) = 0.01801528 kg/mol)—a physical constant used to convert molar flux density to mass flux	kg/mol
cons2	1/specific gas constant of water vapor *R_w_T*	s^2^/m^2^
cons3	A physical constant to convert mass flux resistance of vapor to molar density flux resistance (0.025)	m^3^/mol
GPP	Net carbon acclimation rate per unit leaf area	mol m^−2^ s^−1^
*k_Z_*	Empirical constant giving the ratio of average distance between minor veins and leaf thickness (≥1)	
*k_SP_*	Leaf shape parameter, proportional to *W/L*	
*L*	Leaf length	m
*LA*	Leaf surface area	m^2^
*l_T_*	Length of terminal vein	m
*M_k_*	Number of xylem conduits of *k*th level vein	
*N_k_*	Number of branches of *k*th level vein, *k* = 0…T	
NPP_leaf_	Net carbon gain of one leaf	mol/s
*r*_0_	Radius of xylem conduit of major vein	m
*r_es_*	Mass flux stomatal resistance of water vapor	s/m
*r_k_*	Radius of xylem conduit of *k*th level vein	m
*R*	Carbon cost per leaf volume	mol m^−3^ s^−1^
*r_T_*	Radius of xylem conduit of terminal minor vein	μm
*r_Xk_*	Xylem resistance of a single *k*th order vein	s/m
*r_Xtotal_*	Total xylem resistance of vein of a single flow pathway	s/m
*T*	Terminal minor vein order, also as the total order of the vein	
*W*	Average leaf width	m
*Z*	Thickness of leaf	m
*ρ_l_*	Leaf density	kg/m^3^
*l*_0_	Length of major vein	m
*l_k_*	Length of *k*th level vein	m
*n*	Branching ratio of vein (*N_k_*_+1_/*N_k_*)	
*γ*	Vein network xylem length scaling parameter: l*_n_*_+1_/l*_n_*	
*β*	Vein network xylem radius scaling parameter (*r_n_*_+1_/*r_n_*)	
*m*	Xylem conduits branching ratio *M_k_*_+1_/*M_k_*	
*J*	Diffusive liquid water flux in mesophyll per unit leaf area (flow rate in mesophyll)	m^3^ s^−1^ m^−2^
*Jx*	Liquid water flux per unit area of cross section of conduits (flow rate in conduits)	m^3^ s^−1^ m^−2^
Ψ*_P_*	Water potential of xylem at petiole	Pa
Ψ*_T_*	Water potential of xylem of terminal vein, here we assume the variation of water potential along terminal vein is very small and can be ignored	Pa
Ψ*_C_*	Critical xylem water potential air bubble will form permanently in xylem	Pa
Ψ*_loss_*	Total loss of xylem water potential from petiole to terminal minor vein	Pa
ΔΨ*_L_*_max_	Maximum xylem water potential a leaf can lose from petiole to terminal minor vein	Pa
*E*	Flux of water vapor from inner leaf space to atmosphere through stoma and boundary layer of leaf per unit leaf area	kg m^−2^ s^−1^
*μ*	Dynamic viscosity of water at 25°C (8.90 × 10^−4^ Pa s)	kg m^−1^ s^−1^
*ρ_w_*	Density of water	km/m^3^
*e_sat_*	Saturated water vapor pressure/concentration, a constant, assume air temperature is 25°C	Pa
*e_l_*	Water vapor pressure/concentration within leaf space	Pa
*e_a_*	Partial pressure water vapor of air at 25°C	Pa
*w*_0_	Saturated water concentration in mesophyll, a constant	mol/m^3^
*w*_1_	Water concentration at the evaporative mesophyll surface	mol/m^3^
*D_m_*	Diffusivity of water in mesophyll	m^2^/s
*k_b_*	Physical constant	s^0.5^ m
*d_k_*	Distance between two adjacent *k*th order veins	m
*d_T_*	Distance between two adjacent terminal minor veins	m

Here, using the link between leaf shape and venation, we examine the effect of leaf width‐to‐length ratio and leaf thickness on the coadjustment of the optimal stomatal resistance and leaf area with petiole xylem water potential and corresponding NPP_leaf_ (as NPP_leaf_ per unit of leaf area). For this purpose, we allow leaf width‐to‐length ratio, leaf thickness, and petiole xylem water potential to vary, while treating the rest of the parameters of the leaf model as constant and set using the middle values of the realistic range across different species for biological parameters and the global annual average values for the environmental parameters (Table 2). In addition to NPP_leaf_, we also examine the effect of leaf shape on the change of maximum net carbon assimilation rate, GPP, with ΔΨ*_L_*_max_ (or Ψ*_P_* if Ψ*_C_* does not change). GPP is usually measured in other studies; we include GPP so that we can compare our model's prediction of GPP with other studies.

**Table 2:** Parameters of the leaf model

Symbol	Description	Units	Value	Source
*a*_1_	Plant biochemical parameter, in case of Rubisco limited photosynthesis, it is the maximum carboxylation capacity (*V_c_*_,max_ or *V_m_*)	mol m^−2^ s^−1^	60 × 10^–6^	Katul et al. (2010)
*a*_2_	Biochemical constant	mol/mol	510 × 10^–6^	Katul et al. (2010)
*C_a_*	CO_2_ concentration of air (ambient CO_2_ concentration)	mol/mol	0.0004	Buckley and Schymanski (2014)
	The ratio of resistance of CO_2_ to H_2_O		1.6	
cons2	Inverse of the specific gas constant of water vapor	s^2^/m^2^	7.27 × 10^–6^	
cons1	Molar mass of H_2_O	kg/mol	0.01801528	
cons3	Physical constant to convert mass flux resistance of H_2_O to mol concentration flux resistance at 25°C	m^3^/mol	0.025	Jones (2013, Eq 3.24a)
*D_e_*	Water vapor diffusivity in air	m^2^/s	24 × 10^–6^	
*D_m_*	Diffusivity of water in mesophyll	m^2^/s	8.5 × 10^–10^	Noblin et al. (2008)
*e_a_*	Partial pressure water vapor of air at 25°C	Pa	1,584.5	Calculated from relative humidity of 50% at 25°C
*e*_sat_	Saturated water vapor pressure at 25°C	Pa	3,169	
*k_Z_*			1	
*R*	Carbon cost per leaf volume (the sum of *R_C_* and *R_R_*)	mol m^−3^ s^−1^	0.9 × 10^–6^	Figure 6.3 in Chapin, Matson, & Vitousek, 2011; O'Leary et al., 2017
*r_T_*	Radius of xylem conduit of terminal vein	m	3 × 10^–6^	Blackman et al. (2010), Dunbar‐co, Sporck, and Sack (2009)
*l_T_*	Terminal minor vein length	m	150 × 10^–6^	we take as half of the leaf thickness
*w*_0_	Saturated water concentration in mesophyll	mol/m^3^	40	Noblin et al. (2008)
*Z*	Thickness of leaf	m	300 × 10^–6a^, 250 × 10^–6^, 350 × 10^–6^	Niinemets (1999), Noblin et al. (2008)
Ψ*_C_*	Critical xylem water potential	Pa	−5 × 10^6^	Scoffoni et al. (2017)

Note

a: default value. We use mean leaf area 16 cm^2^ in any conversion between the parameter values of the whole leaf and per leaf area

NPP_leaf_ is maximized when it is equally limited by the loss of xylem water potential from stomatal resistance and total xylem resistance reflected by leaf area (Equation 2), where the total loss of xylem water potential equals the maximum allowable loss of xylem water potential, ΔΨ*_L_*_max_. This can be expressed mathematically in the form of partial differentials of the production function, NPP_leaf_, and the constraint function, Ѱ_loss_, regarding *r*_es_ and *LA*, correspondingly:

(6a) $$\frac{\;\frac{\partial {\text{NPP}}_{\text{leaf}}}{\partial r_{\text{es}}}\;}{\frac{\partial \Psi_{\text{loss}}}{\partial r_{\text{es}}}}=\frac{\;\frac{\partial {\text{NPP}}_{\text{leaf}}}{\partial LA}\;}{\frac{\partial \Psi_{\text{loss}}}{\partial \text{LA}}}$$

(6b) $$\Psi_{\text{loss}}-\Delta \Psi_{L\max}=0$$

By solving Equation (6a), (6b), we can obtain maximum NPP_leaf_ *,* as well as the corresponding optimal LA, and *r*_es_, for any given ΔΨ*_L_*_max._ Assuming Ѱ*_C_*, a plant hydraulic trait, does not change for a given plant, the change of ΔѰ*_L_*_max_ represents the change of Ѱ*_P_*. The optimization is the average condition over the leaf's life span. For leaves growing at different times or locations on a plant, these conditions can vary. However, for a given leaf, these conditions (the biophysical and environmental parameters) are treated as constant in the optimization search for the solution of Equation (6a), (6b). We use the global average values at 25°C for environmental and biochemical parameters (values given in Table 2).

Equation ((6a), (6b)) is solved numerically using the mathematical software MAPLE 2016 (Maplesoft, a division of Waterloo Maple Inc). The solution expresses the maximum NPP_leaf_, optimal leaf area and stomatal resistance, and corresponding GPP, by a set of biophysical and environment variables, as described in Equation 1.

## 3 RESULTS

### 3.1 Model interpretation—optimization

**Figure 2:**
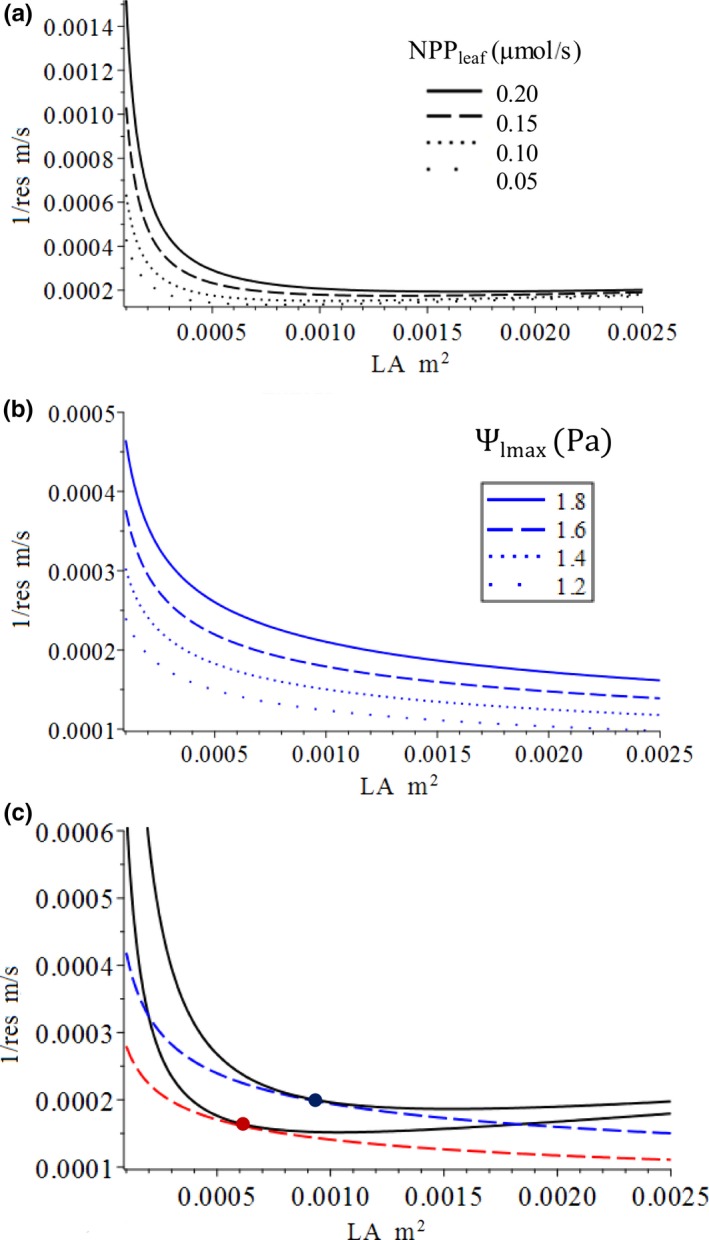
Graphic interpretation of the optimization model: (a) isolines of objective function (Equation 1); (b) isolines of constraint function, and (Equation 2); (c) coadjustment of stomatal resistance and leaf area to maximized net leaf carbon gain when the level of the constraint function increases from a low level (red dash line) to a higher level (blue dash line)

To help in understanding why the model predicts certain patterns, we first interpret the optimization model graphically (Figure 2). Both the objective function (Equation 1) and constraint function (Equation 2) contain three variables: two independent variables—stomatal resistance, *r*_es_, and leaf area, LA*—*and one dependent variable, NPP_leaf_ (or total loss of xylem water potential Ψ_loss_). The objective function and constraint function can be displayed on the graph with isolines (contour lines), with each line representing the combination of *r*_es_ and *LA* that will yield the same NPP_leaf_ (or Ψ_loss_). The form of the equations of the objective and constraint functions determines at which the shape of the isolines the leaf traits and environmental conditions can affect the shape of the isolines. The stomatal conductance, 1/*r*_es_, is used to draw isolines so that NPP_leaf_ increases along both the x and y axes. The isolines of both the objective function (Figure 2a) and the constraint function (Figure 2b) move northeast with increasing level. For a given level of total xylem water potential that a leaf can lose (e.g., the red dashed line), the highest reachable level of NPP_leaf_ is the isoline of the production function that touches the isoline of the constraint function (red dot). A leaf can only produce on and below the isoline of that ΔΨ*_L_*_max_, because above that line, the xylem water potential of terminal minor will be lower than the turgor loss value, and the leaf will be malfunction and no longer be able to properly perform photosynthesis. Setting a lower constraint (e.g., moving from the red to blue line) increases both stomatal conductance and leaf area, as well as NPP_leaf_ (blue dot) (Figure 2c). This explains the general trends. The parameters of the model (Equation 2) affect the shapes of the isolines of the two functions. With different values, the exact location of the optima varies. Next, we discuss the effect of leaf shape as parameters on the leaf optima.

### 3.2 Impact of leaf shape‐venation relation on leaf optimum

**Figure 3:**
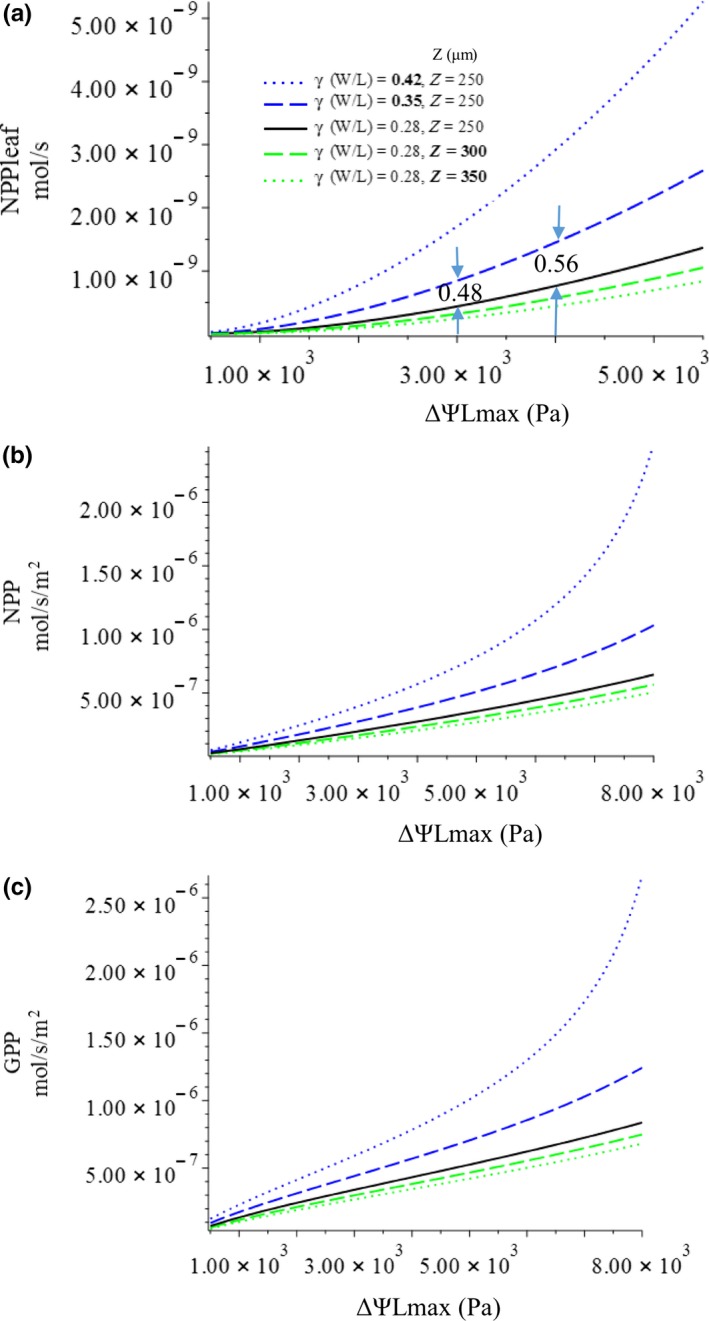
Change of (a) net leaf carbon gain (NPP_leaf_); (b) net leaf carbon gain rate per unit leaf area (NPP); and (c) net carbon assimilation rate per unit leaf area (GPP) with total allowable loss of xylem water potential

**Figure 4:**
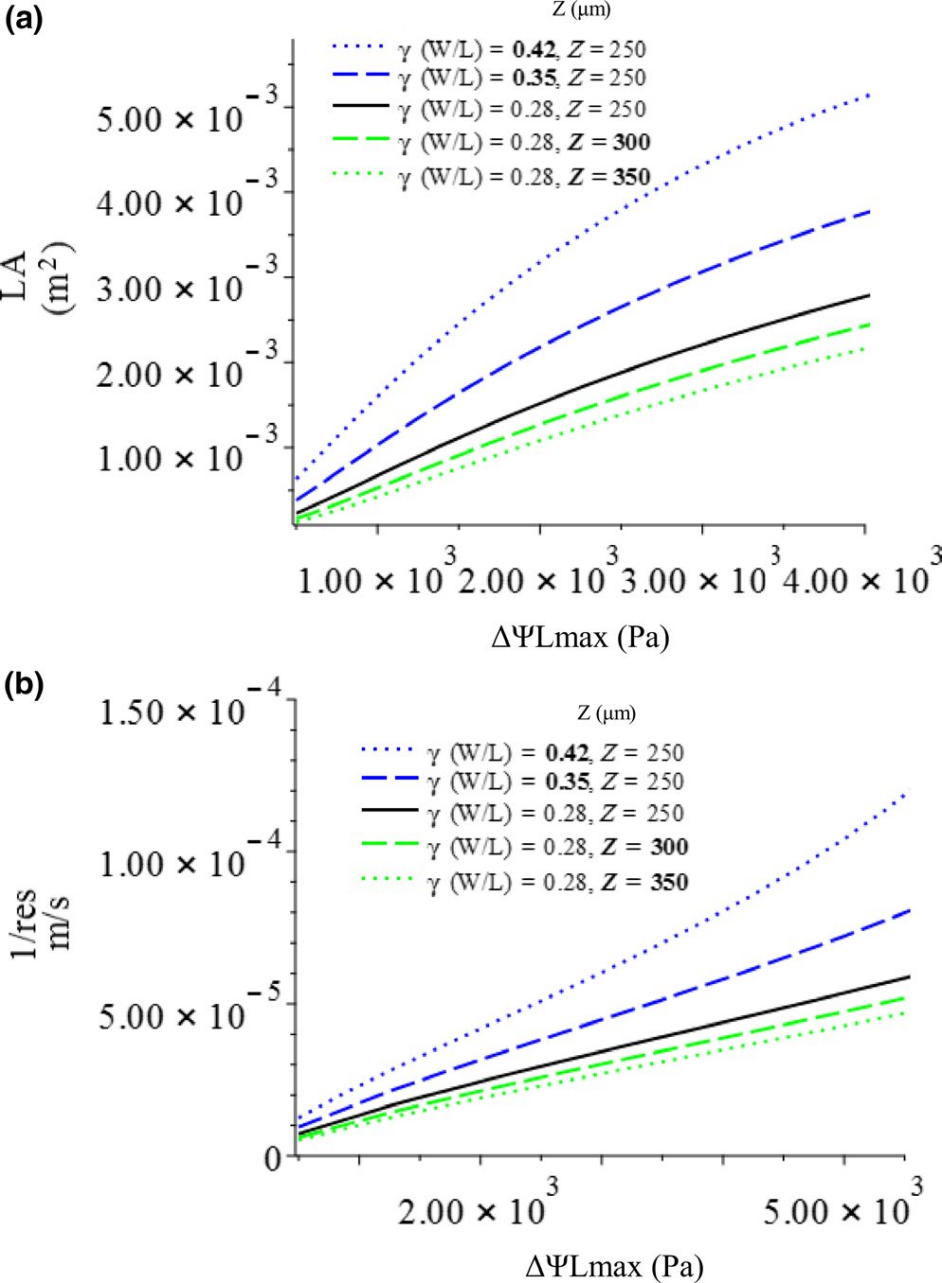
Change of (a) optimal leaf area and (b) stomatal resistance with increasing allowable loss of total xylem water potential

First, we examine the effect of leaf width‐to‐length ratio on the change of leaf optimum, GPP, and NPP_leaf_ with increasing petiole xylem potential (Figures 3 and 4, blue lines). Our model predicts that the GPP and NPP_leaf_ of leaves will increase with different *W/L* ratios, with the allowable loss of total xylem water potential (ΔΨ*_L_*_max_; Figure 3); the GPP and NPP_leaf_ of a narrowleaf (small *W/L*) will, overall, be lower than that of a broadleaf (large *W/L*). The marginal effect of increasing leaf W/L ratio on GPP, *NPP*, and NPP_leaf_ increases with ΔΨ*_L_*_max_. For example, increasing *W/L* from 0.28 (solid black line in Figure 3a) to 0.35 (blue dashed line in Figure 3a) at ΔΨ*_L_*_max_ = 3x10^3^ Pa, NPP_leaf_ increases by about 0.47 mol/s, but at ΔΨ*_L_*_max_ = 4x10^3^ Pa, NPP_leaf_ increases by about 0.65 mol/s. Furthermore, both the GPP and NPP_leaf_ of a narrowleaf increase with ΔΨ*_L_*_max_ at a much slower rate than those of a broadleaf (Figure 3). This means that a broadleaf is more sensitive to the change of ΔΨ*_L_*_max_ and will have higher marginal gain of productivity than a narrowleaf. However, on the other hand, if ΔΨ*_L_*_max_ decreases, a broadleaf will also suffer greater productivity loss than a narrowleaf.

The optimal leaf sizes of narrow and broad leaves will increase with increasing ΔΨ*_L_*_max_, but that of a narrowleaf will increase with ΔΨ*_L_*_max_ more slowly than that of a broad leaf; also, the optimal leaf size of a leaf with a small *W/L* will, overall, be smaller than that of a leaf with a large *W/L* (Figure 4a). The optimal stomatal conductance of leaves with different *W/L* ratios will increase with increasing ΔΨ*_L_*_max_; the optimal stomatal conductance of narrow leaves will be lower than that of broad leaves at any petiole xylem water potential (Figure 4b). Assuming Ѱ*_C_*, a plant hydraulic trait, does not change for a given plant, the change of total allowable xylem water potential loss (ΔѰ*_L_*_max_) represents the change of petiole xylem water potential (*Ѱ_P_*); the result of the optimization shows that the GPP, NPP_leaf_, and optimal leaf area will all increase with petiole xylem water potential, while stomatal resistance will decrease, each at different rates. The marginal increases of the GPP and NPP_leaf_ of a narrowleaf will be smaller than those of a broad leaf.

### 3.3 Effect of leaf thickness

The model predicts that with different leaf thicknesses, NPP_leaf_, net carbon gain per leaf area (NPP), GPP, and optimal leaf area will all increase with petiole xylem water potential, while optimal stomatal resistance will decrease (Figures 3 and 4, green lines). However, overall, along the gradient of maximum allowable water potential loss (*ΔѰ_L_*_max_), a thicker leaf will have lower productivities, which also change with ΔѰ*_L_*_max_ at a slower rate (Figure 3, green lines). This will result in a more significant effect of leaf thickness on NPP_leaf_ and leaf optimums when petiole xylem water potential is high. In other words, NPP_leaf_, GPP, optimal stoma resistance, and leaf area are more sensitive to changes of petiole xylem water potential if the leaf is thin. A thicker leaf will also have higher optimal stomatal resistance and lower optimal leaf area than those of a thin leaf, at any given ΔѰ*_L_*_max_ (Figure 4, green lines). The effects of leaf thickness on the above leaf properties are not as strong as the effects of leaf W/L ratio.

## 4 DISCUSSION

### 4.1 Implications of the model

Our leaf model predicts two patterns: (a) stomatal conductance (1/res, inverse of stomatal resistance), leaf area, and GPP increase with increasing petiole xylem water potential; (b) with different leaf shapes, the aforementioned variables change at different rates with petiole xylem water potential. This is due to different venation structures.

Our first prediction agrees with other previous studies on the changing of leaf traits with tree height. These studies, conducted in different places, have found that net carbon assimilation rate and leaf area decrease with tree height due to decreased xylem water potential along the stem (Ambrose et al., 2009; Ishii et al., 2008; Kenzo et al., 2015; Koch et al., 2004; Ryan et al., 2006). Ishii et al. (2008) have found that bulk leaf water potential decreases with the height of the leaf for *Sequoia sempervirens*. They have also found that leaf area decreases from ~9–1 cm^2^ from the bottom to top of the tree (Figure 3d in Ishii et al., 2008) and that net carbon assimilation rate first increases with height as a result of increasing light intensity, reaching the maximum at 80 m, then decreases with height as the xylem water potential decreases with height along the tree. Stomatal conductance also decreases with height from 250 mm mol m^−2^ s^−1^ at the base to 800 mm mol m^−2^ s^−1^ at the top of the tree (Figure 5c in Niinemets, 2002). Koch et al. (2004) have found that xylem water potential decreases with height linearly. Leaf size and shape also change with height: leaves become smaller and more expanded. Using carbon isotopes, Koch et al. (2004) have confirmed that the reduction in photosynthesis rates with height is caused by the reduced stomatal conductance. These studies support the argument that leaf morphology and stomatal conductance are equally limited by leaf water status (Koch et al., 2004; Niinemets, 2002; Thomas & Winner, 2002; Woodruff, Bond, & Meinzer, 2004; Woodruff, Meinzer, Lachenbrunch, & Johnson, 2009).

Furthermore, our model suggests that the properties of plant hydraulic systems may affect stoma response. Within xylem, water flows through tracheids or vessels, tube‐like dead cells (conduits), from petiole to minor vein. The hydraulic conductivity of xylem is determined by the inner conduit lumina, which is related to the number of pits and its anatomy, the end of conduit connection, and the length and width of the conduits. Water flow in the xylem is treated as pipe flow in our model. In a pipe model, the conductivity of a pipe is determined by the radius of the pipe, which is the representative conduit radius (*r_T_*) in our model. The effect of the above‐mentioned conduit properties on xylem hydraulic conductivity can be generalized through this parameter.

Based on current knowledge, water flows from xylem to spongy mesophyll cells through the bundle sheath made by parenchymatous cells and then evaporates at the mesophyll surface (Sack and Holbrook, 2006). The structure and anatomy of the cells of bundle sheath and mesophyll determine the resistance of outside xylem water flow. The variation of these properties can be captured by the parameter of mesophyll diffusivity, *D_m_*, in our model. For example, increasing the cell wall thickness of a bundle sheath can be represented by a lower *D_m_*.

Empirical study has found the general pattern that species having higher maximum stomatal conductance quickly close their stoma at low vapor pressure deficit (VPD; Oren et al., 1999). Recently, Henry et al. (2019) have developed a stoma response model that explains this pattern through four mechanisms. The optimal stomatal resistance in our model is equivalent to the maximum stomatal conductance, or gsmax. Our model can be connected to their stoma model through optimal stomatal resistance to further investigate the effect of hydraulic traits, such as mesophyll resistance and pit property, on the instantaneous stoma response to VPD, through the parameters *r_T_* and *D_m_*.

Our second prediction is based on the assumption of the relation of leaf shape and hydraulic systems. Next, we discuss in detail the connection between leaf shape and hydraulic conductance through the regulation of vein structure.

### 4.2 Effect of leaf shape on leaf hydraulic conductance

**Figure 5:**
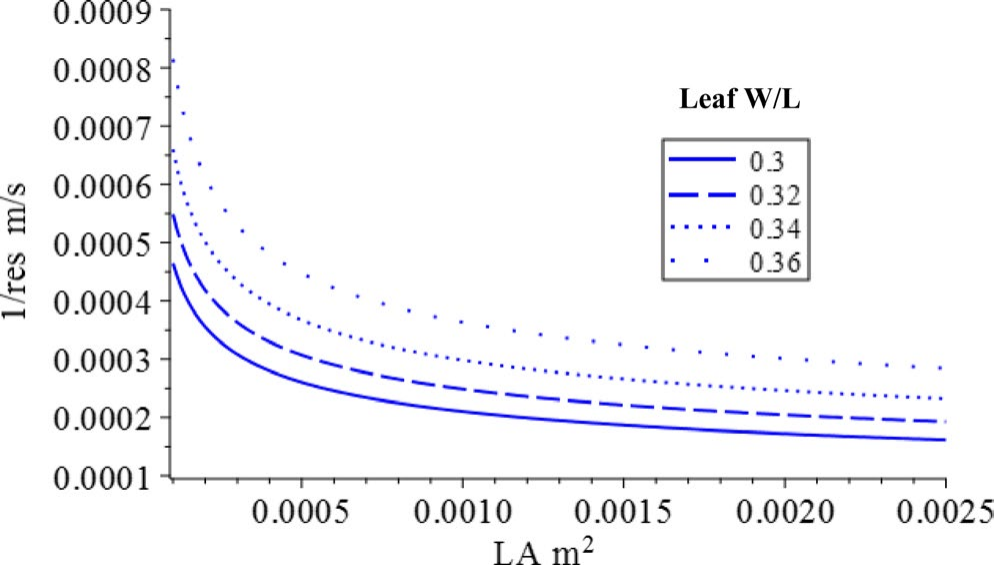
Effect of leaf width‐to‐length ratio on the isoline line of the constraint function. All four lines represent the same level of total loss of xylem water potential. With larger *W*/*L* ratio, the isoline line (dot line) is located in the northeast; it migrates southwest as the *W*/*L* ratio decreases

**Figure 6:**
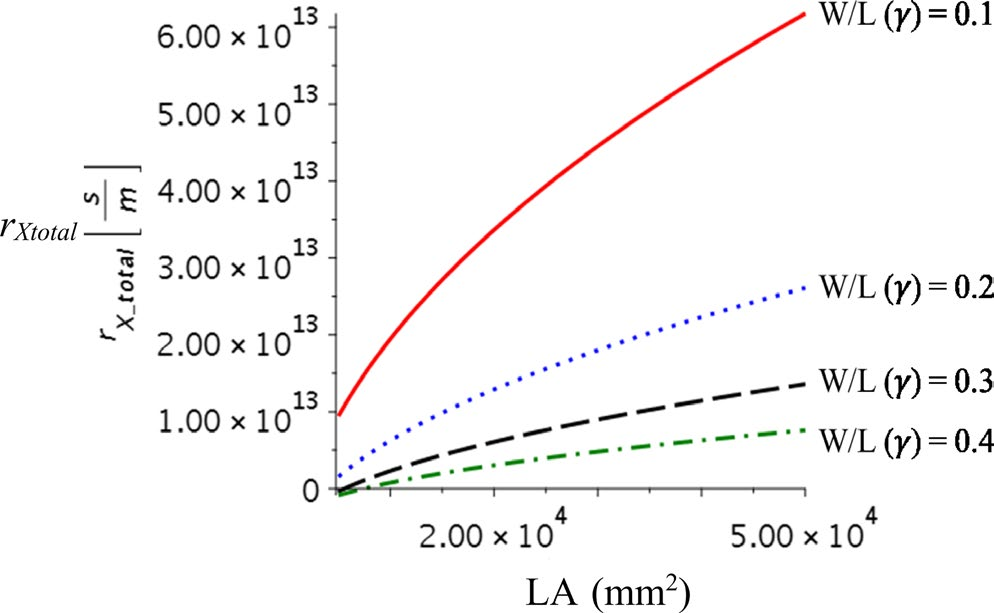
Change of total xylem resistance with leaf area of different leaf shape (*W*/*L* ratio), lines represent different *W*/*L*

Leaf shape affects NPP_leaf_, optimal stomatal resistance, and leaf area through the vein network structure parameters of the constraint function—*γ* and *β* within our leaf model—and leaf width‐to‐length ratio determines the leaf hydraulic network parameter *γ* (i.e., the length ratio of daughter‐to‐mother conduits [and veins]). This parameter determines the rate at which total xylem resistance *r*_Xtotal_ scales with leaf area. Increasing *W/L* ratio causes at the same level Ψ_loss_ isoline to move northwest (Figure 5). This means that given the same amount of water potential that can be lost, a leaf with a larger *W/L* ratio can support higher carbon production factors, stomatal conductance, and leaf area than a leaf with a smaller *W/L* ratio (Figure 5). This occurs because the rate at which the total xylem resistance increases with leaf area is much faster when *γ* is smaller (Equation 2, Figure 6). This causes the narrowleaf to have smaller optimal leaf area but higher optimal stomatal resistance than those of a broadleaf and thus a lower maximum NPP_leaf_. The stomatal resistance of a narrowleaf is consequently higher than that of a broadleaf with the same leaf area.

**Figure 7:**
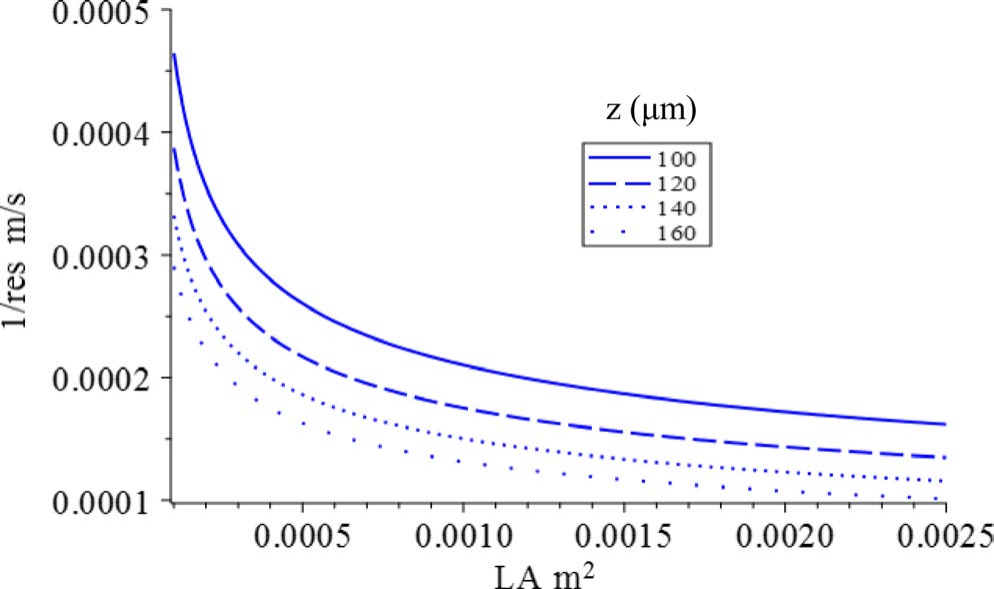
Effect of leaf thickness on the location of the isoline line of the constraint function. The isoline lines have the same level of total loss of xylem water potential, but with different leaf thickness, *Z*, they differ in location and curvature

It should be noted that the minor vein density controls the relation between the covariance of stomatal conductance, leaf area, and total xylem water potential loss. Leaf thickness affects optimal stomatal conductance, leaf area, and NPP_leaf_ if the minor vein distance is proportional to leaf thickness, as observed in angiosperms (Noblin et al., 2008). In our model, minor vein density is linked to leaf thickness through the term $k_{Z}\cdot Z/2$, which represents the distance between the two adjacent minor veins (i.e., the inverse of minor vein density). For a given level constraint, increasing minor vein distance not only shifts the isoline of the constraint function inward but also increases its curvature (Figure 7). This means that given the same amount of total xylem water potential loss, increases in the distance between minor veins (decreases in minor vein density) will result in lower stomatal conductance and smaller leaf area, which consequently lowers the maximum NPP_leaf_ that can be reached. For plants whose minor vein density is independent of leaf thickness, leaf thickness will have no effect on the leaf optima or NPP_leaf_ from a hydraulic perspective. However, increasing leaf thickness may result in higher respiration costs and thus have a negative impact on NPP_leaf_.

The physiological explanation of our model is that with the same amount of transpiration per unit leaf area (e.g., with the same stomatal conductance per unit leaf area), a thicker leaf with lower minor vein density will require increasing the flow rate in the conduit, thus causing more loss of total xylem water potential. This effect is greater when petiole xylem water potential is high.

Increasing leaf thickness by increasing volume of mesophyll can decrease leaf mass per unit area (LMA; Enrique, Olmo, Poorter, Ubera, & Villar, 2016; Griffith, Quigley, & Anderson, 2016). Our model predicts that a thick leaf will also have higher stomatal resistance, which will result in lower maximum carbon assimilation rate per unit leaf area (and per unit leaf mass); therefore, a negative relation between LMA and maximum carbon assimilation rate results from changing leaf thickness. This negative relation between LMA and maximum carbon assimilation rate has been observed worldwide (Wright et al., 2017).

### 4.3 Linking the leaf to the whole plant and landscape

The individual leaf is the fundamental unit of a whole tree. Understanding the carbon balance of an individual leaf is the first step and is critical for understanding the whole plant's carbon balance. In this study, we have connected vein structure and leaf shape, which is further incorporated into the coupling of the transpiration and photosynthesis processes. This has provided us a to upscale biophysical processes occurring at the stoma scale to whole plants and, further, to the landscape.

Leaf shape and area also affect the light penetration and distribution of the canopy (Bonan, 1996; Dickinson, 1983; Sellers, 1985). Smith, Sperry, and Adler (2016) have developed a model of optimizing the light usage of a tree. The relation between leaf area and number of leaves is more relavent to light utilization. This further affects the architecture of the crown to maximize light utility, which is achieved by regulating the stem branching network structure. Through this mechanism, our model can be coupled with Smith et al.'s model (2017) and the light utility and crown architecture models (Johnson, Smith, Vogelmann, & Brodersen, 2005; Niinemets & Anten, 2009) to scale from leaf to whole plant. Our model presents a possible method to connect the internal and external anatomy of a leaf to the leaf water status. This provides a means to couple leaf boundary layers, vapor pressures, and stomatal conductance to investigate the feedback between these traits in terms of the leaf carbon budget.

The hydraulic safety–efficiency trade‐off—namely, plants whose vascular systems are more resistant to cavitation but inefficient at water transport—is a widely recognized phenomenon (Hacke, Sperry, Wheeler, & Castro, 2006; Manzoni et al., 2013; Markesteijn et al., 2011; McElrone, Pockman, Martínez‐Vilalta, & Jackson, 2004). It is found that, in general, species with lower xylem conductance have safer xylem (Venturas et al., 2017). Both hydraulic traits (pit and xylem network properties), and nonhydraulic traits (e.g., wood density) can contribute to the hydraulic safety–efficiency trade‐off (Gleason et al., 2016). Among the hydraulic traits, pit properties have strong effects on the safety of xylem, while conduit diameter is a xylem network property found to be related to large P50 in xylem (safer xylem) in some species (Blackman, Brodribb, & Jordan, 2010; Brodribb, Bienaimé, & Marmottant, 2016; Scoffoni et al., 2017). For example, Blackman et al. (2010) have found the P50 in the leaves of 20 phylogenetically disparate woody angiosperm species from montane rainforest and dry sclerophyll forest in cool, temperate Australia to be significantly correlated with the average lumen width of the vein. In our model, a narrowleaf has a small *γ* (the length ratio of two consecutive veins) and a small xylem conduit diameter and thus a higher total xylem resistance compared to a broadleaf with the same leaf area. Following the scaling relationship in WBE theory (West et al., 1997), small xylem conduits located at the end of the petiole will result in smaller xylem conduits throughout the entire vein network along the whole plant. Thus, plants with narrow leaves will have smaller xylem conduits and lower hydraulic(xylem) conductivity and thus be less efficient in transporting water. On the other hand, plants with broad leaves will have larger xylem conduits and higher hydraulic (xylem) conductivity. Whether large conduits reduce the resistance of xylem to cavitation or not, assuming that the hydraulic safety–efficiency trade‐off does exist, having broader leaves will cause plants to be more vulnerable to cavitation.

**Figure 8:**
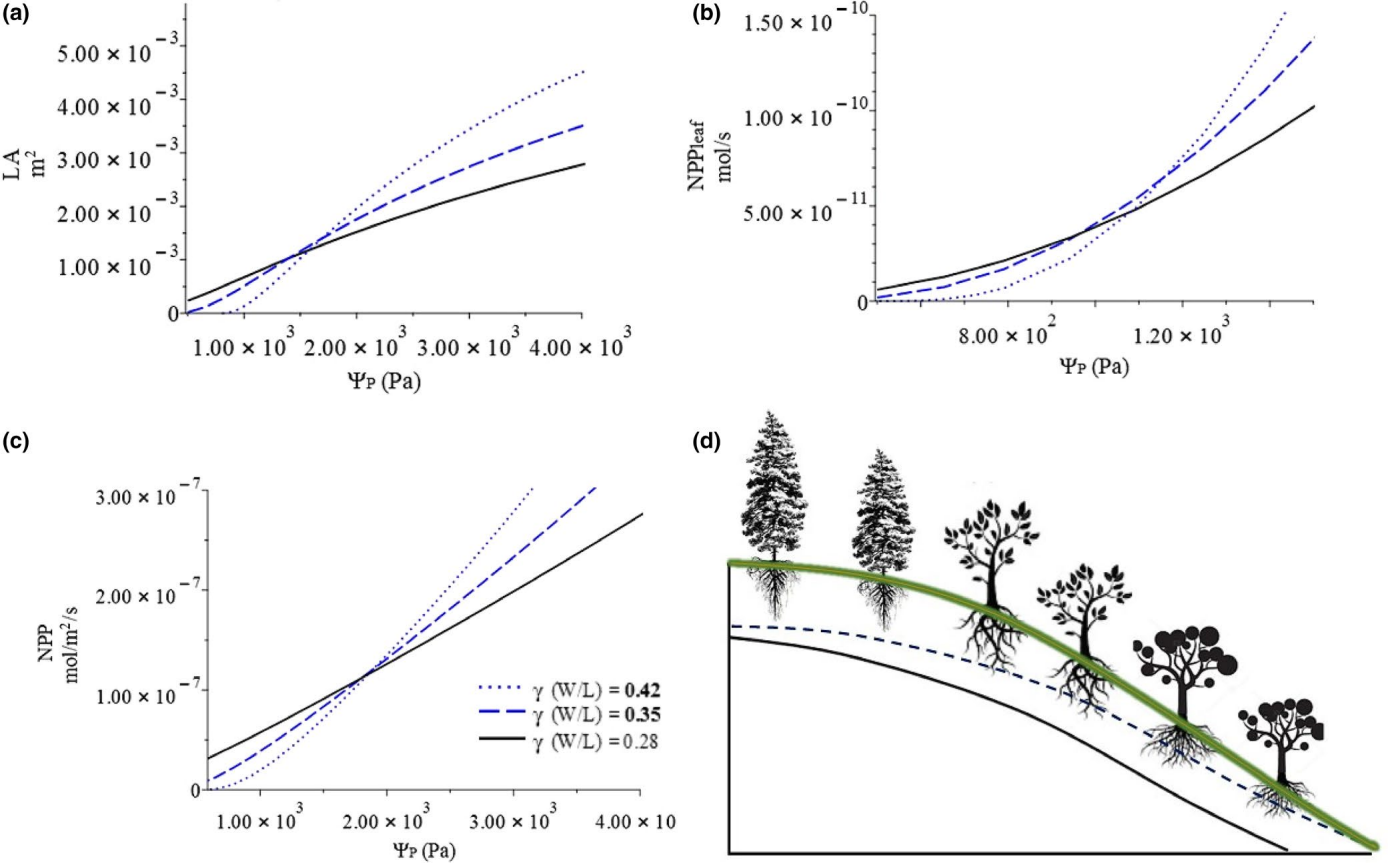
Theoretical change of (a) leaf area, (b) net leaf carbon gain, and (c) net leaf carbon gain per unit leaf area with petiole xylem water potential due to safety–efficiency trade‐off of plant hydraulic system and potential impact on plant distribution along hillslope from topographically induced soil moisture gradient (d)

Inefficiency in water transport also implies inefficiency in carbon fixation; thus, a narrowleaf plant will have an advantage when soil moisture is low but gradually lose that advantage with increasing soil moisture. So, the net primary productivities of narrowleaf plants increase with soil moisture more slowly than those of broadleaf plants. As a consequence, with their size increasing more slowly with increasing soil moisture, narrowleaf plants will gradually lose their advantage to broadleaf plants in wetter soils (Figure 8b, c).

Soil moisture is regulated by topography (Barling, Moore, & Grayson, 1994; Beven & Kirkby, 1979; Iorgulescu & Musy, 1997; O'Loughlin, 1986); it is lowest at ridge top and increases along the flow path, moving down the hillslope toward the channel. Thus, in general, along the hillslope, narrowleaf plants dominate the ridge area where soil moisture is low. Moving down along the hillslope, the soil becomes wetter, and broadleaf plants become more dominant (Figure 8d).

Recently, the recognition of the interaction between plants and earth surface processes has led to interdisciplinary studies of the coevolution of plants and their physical environments (Corenblit, Steiger, Gurnell, & Naiman, 2009; Fisher, Heffernan, Sponseller, & Welter, 2007; Murray, Knaapen, Tal, & Kirwan, 2008; Reinhardt, Jerolmack, Cardinale, Vanacker, & Wright, 2010). However, an appropriate means to explicitly integrate the feedback mechanism between plant and earth surface processes remains unclear. The leaf model here can further facilitate these studies. Ding, Johnson, & Martin (2018) have developed a formula that can be used to directly connect groundwater regulated soil moisture to advection and diffusion erosion processes (i.e., landscape development processes) in low order watersheds. Once the leaf model is scaled to the whole plant and integrated into the advection and diffusion landscape processes, we can predict the abundance and distribution of a given plant throughout the landscape using the two erosion coefficients and the leaf shape.

## 5 CONCLUSION

We have connected leaf vein structure to leaf shape, which has provided the scaling relation of the total xylem resistance of veins with leaf area. This relation has been incorporated into the coupling of the transpiration and leaf carbon budget of a leaf model to investigate the impact of leaf shape on how plants coadjust stomatal resistance and leaf area to maximize NPP_leaf_. The optimality model predicts that both NPP_leaf_ and leaf area increase with petiole xylem water potential, while stomatal resistance decreases, each at different rates. A narrowleaf has an overall lower NPP_leaf_ and leaf area but higher stomatal resistance compared to a broad leaf. This occurs because a broadleaf has a larger vein length ratio, γ as suggested by our model. With the same leaf area, a broadleaf will have, on average, larger xylem conduits and consequently a more efficient transportation of water and less resistance to cavitation than a narrowleaf. Our study indicates that when incorporating the impact of vein structure on the vulnerability curve, a trade‐off exists between a higher marginal leaf carbon fixing rate with respect to xylem water potential and the ability of the leaf to resist cavitation. This explains how plants adapted to dry and/or hot conditions will gradually lose their advantages with increasing soil moisture. Using the link between vein structure and leaf shape, we can further connect plant hydraulic traits to other traits affecting strategies of leaf thermal regulation and canopy light utility, thereby providing a method to upscale from stoma to whole plants and the ecosystem.

## CONFLICT OF INTEREST

The authors declare no conflicts of interest.

## AUTHOR CONTRIBUTIONS

J.D. created the model and performed the analysis. J.D., E.J., and Y.M. wrote the paper. E.J. provided funding.

### Open research badges

This article has earned an Open Data for making publicly available the digitally‐shareable data necessary to reproduce the reported results. The data is available at https://github.com/JunyanDing/Leaf-Optimzation-Model-

## Data Availability

Model input file is available at https://github.com/JunyanDing/Leaf-Optimzation-Model-
