# Supplementary material for: Optimization of leaf morphology in relation to leaf water status: A theory
Source: Ecol Evol. 2020 Jan 22;10(3):1510–25. doi: 10.1002/ece3.6004 (PMC7029057; doi:10.1002/ece3.6004)
Supplement: Supplementary file 1 [file ECE3-10-1510-s001.docx]

## Appendix – derivation of the equations

The supporting material describes the steps of the derivation of the main equations of the constraint function in the main text

**S1. Derivation of Equation 2a, 6 and 7**

Following Price and Enquist (2007), we assume that the conduits contained by vein branch where the vein branches, the length of *k*th order conduit is the same as the length of kth order vein. But, the branching ratio of conduits and veins are different, Noticing, in addition to these assumption, our leaf model also has two additional assumptions: 1) the length ratio of two consecutive veins, γ, is proportional to half of the width to length ratio of the leaf; 2)the distance between two adjacent terminal minor veins is half of the leaf thickness (Figure 1). Based on these assumptions, we define:


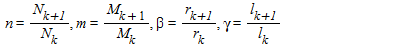


where *n* is branching ratio of vein, *m* is the branching ratio of xylem conduit, *N_k_* is the number of *k*th order vein, *M_k_* is the number of conduits contained by *k*th order vein, *r_k_* and *l_k_* are the radius and length of *k*th order conduit, *β* is the ratio of the radius of the conduits of two consecutive vein, *γ* is the ratio of the length of the conduits of two consecutive vein. The order of vein is defined in the way that major vein is in order 0, secondary major vein is in order 1, and so on. This gives:


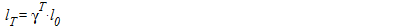
(S1)

where *T* is the order of terminal minor vein, which is also the highest order. Since 0th order vein is the major vein, thus *l_0_*=*L*, (*L* is the length of leaf) thus T is give by:
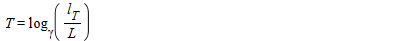
 (S2)

Given the steady laminar flow of a Newtonian fluid, using Poiseuille formula, the total xylem resistance of *k*th order vein is:


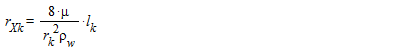
 (S3)

where μ is dynamic viscosity of water (kg m^-1^ s^-1^), ρ_w_ is the density of liquid water (kg m^-3^), The total xylem resistance of the vein of a single flow path is:


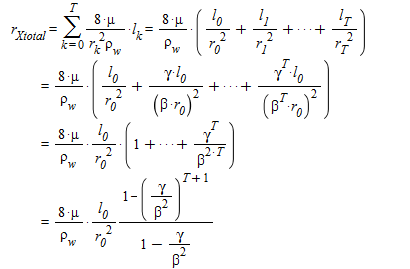
 (S4)

Since *l_0_*= *l_T_* x *γ^-T^*, and *r_0_*=*l^T^* x *β^-T^*, we can write Equation S4 as


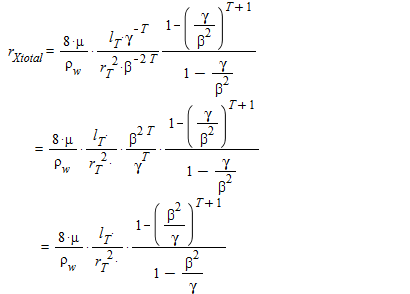
(S5)

Substitute *T* by Equation S2, Equation S5 becomes:


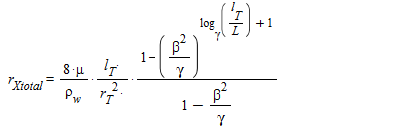
(S6)

By rearranging Equation S6 the total xylem resistance of a single flow path can be written as the power low function of total leaf length, where the xylem network structure parameters (*l_T_, r_T_, γ, and β*) determine the exponent and the slope coefficients:


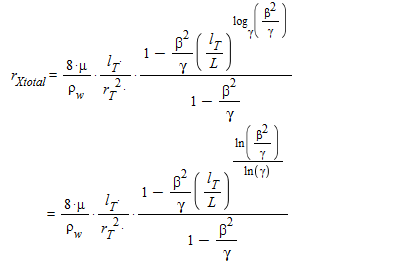
(S7)

Note, the water flow through the xylem network in form of liquid water but the diffusion is in form of vapor. Here, we convert the vapor flux into liquid form of mass (kg s^-1^ m^-2^) in order to make consistence. In all the equations in the derivation and in the paper, the flux of water is presented as liquid form. The constants, cons1 and cons2 are physical constant (see table 1 in MS for the values) for converting molar mass to mass and vapor flux to liquid flux in the derivation below.

The total drop of the xylem water potential along the single flow pathway from petiole to terminal minor vein is:

$\Psi_{loss}=J_{x}r_{Xtotal}$ (S8)

where *J_x_* is the average mass flow rate per unit area of cross section (kg s^-1^ m^-2^). For an energy conservation system – a system that will minimize the energy loss of the transport system, *J_x_* is constant along the flow path (West et al., 1999). At equilibrium (equal volume), the total volume of water moves through the total cross-section of each level of vein equals total transpiration rate of the leaf, which is the transpiration rate per unit leaf area, E, multiplied by leaf surface area. This gives:


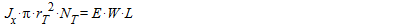
(S9)

*E* is the transpiration rate per unit leaf area as mass (kg s^-1^ m^-2^).

Assume the vein network is space filling, thus:


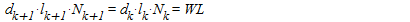
(S10)

where *d_k_* is the distance between two adjacent veins of order *k*, *l_k_* is the length of *k*th order vein and *N_k_* is the number of *k*th order vein. As for minor veins, we can write:


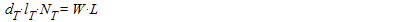
(S11)

This gives the number of minor veins as:


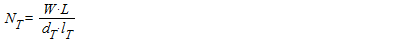
(S12)

If the distance between two adjacent minor veins is larger than the distance from the minor vein to evaporative surface, the total flow flux in form of liquid water through mesophyll per unit leaf area is (modified from Equation 1 in Noblin et al. 2008):


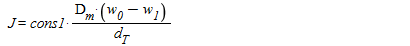
(S13)

where *cons1* is the molar mass of H_2_O (M(H_2_O)=0.01801528 kg/mol), *D_m_* (m^2^ s^-1^ ) is the mesophyll diffusivity given by Noblin et al. (2008) (note the diffusivity of messophyll depends on the molecular structure of the messophyll cells and their arrangememnt the plant species, but examine the effect of mesophyll structure is beyond the scope of this study thus we use the value given by Noblin et at. 2008). *w*_0_ (mol m^-3^) is the water concentration of mesophyll surface touching the minor vein surface, *w*_l_ (mol m^-3^) is the water concentration at evaporative mesophyll surface. Note, Jx and J are different, with Jx refers to the flux per unit cross section of vein conduit and J refers the flux through mesophyll per unit leaf area. E below refers to flux as transpiration rate per unit leaf area.

If the distance between two adjacent minor veins is much smaller than that between the minor vein to evaporative surface (*h*), the total flow rate per unit leaf area is (modified from Equation 2 in Noblin et al. 2008):


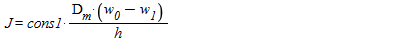
(S14)


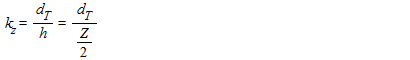
We define a new parameter *k_z_* as the ratio of minor vein distance to leaf thickness:

(S15)

We can write Equation 11 and 12 in one general form:

$J=cons1\cdot\frac{D_{m}\cdot(w_{0}-w_{1})}{k_{Z}\cdot h}$ (S16)


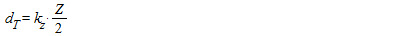
Assuming that the surface mesophyll is close to leaf surface so that the diffusion of vapor from mesophyll to stoma can be ignored, the optimal distance between two terminal minor vein is half of the leaf thickness (Noblin et al. 2008). Thus, h is approximately to half of the leaf thickness, Z/2 and , Equation S16 becomes

$J=cons1\cdot\frac{D_{m}\cdot(w_{0}-w_{1})}{k_{Z}\cdot\frac{Z}{2}}$ (S17)

This also gives:


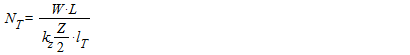
 (S18)

So as for most of the angiosperms, the minor vein density is approximately half of the leaf thickness, thus *k_z_* equals 1, and for needle leaf species, *k_z_* >1 and can vary depends on the species. The relation between w_l_ and w_0_ (Randau and Doyle, 2005; Noblin et al., 2008) is given as:


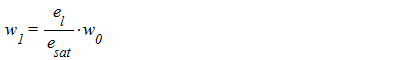
 (S19)

*e_l_* (Pa = kg m^-1^ s^-2^) is the partial vapor pressure of inner leaf space, and *e_a_* is the partial vapor pressure of air. Substitute *w_l_* in Equation S17 by Equation S17, we get

 (S20)


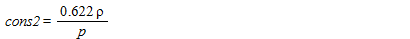
The diffusion rate of water vapor from inner leaf space to atmosphere is:


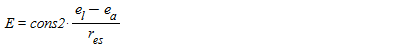
, (S21)

where *E* is the transpiration flux in form of liquid water (kg m^-2^ s^-1^) where *p* is average air pressure at sea level (101.325 kPa) and *ρ* is air density at 25^o^C (1.255 kg m^-3^), both of which are constants. This gives *cons2* as 7.704 x 10^-6^ (m-^2^ s^2^). *e_l_* (Pa = kg m^-1^ s^-2^) is the partial vapor pressure of inner leaf space, and *e_a_* is the partial vapor pressure of air, and *r_es_* (s m^-1^) is the stomatal resistance of vapor per unit leaf area.

At equilibrium, *E*=*J*. Now we have two equations (Equation S16 and S18) and two variables: *E* (*J*) and $e_{l}$. Solving Equation S20 and S21 together for *E*, we get:

 (S22)

Now we have the equation for transpiration. Next, by substituting *N_T_* by Equation S18 and rearrange the Equation S9, we get:


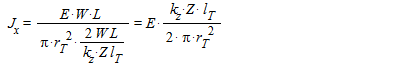
(S23)

Substitute *E* by Equation S22 in Equation S23, we get *Jx*:

 (S24)


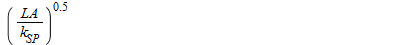
Given *LA*=*L* $\times$ *W*, and *W*=(*W*/*L*) $\times$ *L*, thus *LA*=*L^2^* $\times$ (*W*/*L*), assigning *k_SP_* =*W*/*L*, *L* can be written substituting *L* by this formula in Equation S7, then substitute *J_x_* by Equation S24 and *r_Xtotal_* by Equation S7 in Equation S8, the loss of total xylem water potential from petiole for a terminal minor vein is:

$\begin{matrix} \Psi_{loss}=J_{x}\cdot r_{Xtotal} \\ \text{ }=\frac{e_{sat}-e_{a}}{\frac{\frac{k_{z}\cdot Z}{2}\cdot e_{sat}}{cons1\cdot D_{m}\cdot w_{0}}+\frac{r_{es}}{cons2}}\cdot\frac{k_{z}\cdot Z\cdot l_{T}}{2\pi\cdot r_{T}^{2}}\cdot\frac{8\cdot\mu}{\rho_{w}}\cdot\frac{l_{T}}{r_{T}^{2}}\cdot\frac{1-\frac{\beta^{2}}{\gamma}\cdot\left(\frac{l_{T}}{L}\right)^{\frac{\ln(\beta^{2}/\gamma)}{\ln\gamma}}}{1-(\beta^{2}/\gamma)} \\ =\frac{e_{sat}-e_{a}}{\frac{\frac{k_{z}\cdot Z}{2}\cdot e_{sat}}{cons1\cdot D_{m}\cdot w_{0}}+\frac{r_{es}}{cons2}}\cdot\frac{k_{z}\cdot Z\cdot l_{T}}{2\pi\cdot r_{T}^{2}}\cdot\frac{8\cdot\mu}{\rho_{w}}\cdot\frac{l_{T}}{r_{T}^{2}}\cdot\frac{1-\frac{\beta^{2}}{\gamma}\cdot\left(\frac{l_{T}}{\left(LA/k_{SP}\right)^{0.5}}\right)^{\frac{\ln(\beta^{2}/\gamma)}{\ln\gamma}}}{1-(\beta^{2}/\gamma)} \end{matrix}$ (S25)
